# Cross-Cultural Content Validity of the Autism Program Environment Rating Scale in Sweden

**DOI:** 10.1007/s10803-018-03870-5

**Published:** 2019-01-08

**Authors:** Hampus Bejnö, Lise Roll-Pettersson, Lars Klintwall, Ulrika Långh, Samuel L. Odom, Sven Bölte

**Affiliations:** 1 Department of Special Education, Stockholm University, Stockholm, Sweden; 2 Department of Psychology, Stockholm University, Stockholm, Sweden; 3 Center of Neurodevelopmental Disorders (KIND), Division of Neuropsychiatry, Department of Women’s and Children’s Health, Karolinska Institutet, Stockholm, Sweden; 4 Center for Psychiatry Research, Stockholm County Council, Stockholm, Sweden; 5 Autism Center for Young Children, Habilitation & Health, Stockholm County Council, Stockholm, Sweden; 6 Frank Porter Graham Child Development Institute, University of North Carolina at Chapel Hill, Chapel Hill, USA; 7 Child and Adolescent Psychiatry, Stockholm County Council, Stockholm, Sweden; 8 Curtin Autism Research Group, School of Occupational Therapy, Social Work and Speech Pathology, Curtin University, Perth, Western Australia

**Keywords:** Autism spectrum disorder, Scale, Preschool, Assessment, Cultural adaption, Content validity

## Abstract

Increasing rates of autism spectrum disorder (ASD) and younger age at diagnosis pose a challenge to preschool intervention systems. In Sweden, most young autistic children receive intervention service in community-based preschool programs, but no tool is yet available to assess the quality of the preschool learning environment. This study adapted the Autism Program Environment Rating Scale Preschool/Elementary to Swedish community context (APERS-P-SE). Following translation and a multistep modification process, independent experts rated the content validity of the adaptation. Findings indicate high cross-cultural validity of the adapted APERS-P-SE. The cultural adaption process of the APERS-P-SE highlights similarities and differences between the American and Swedish preschool systems and their impact on early ASD intervention.

The rates of children diagnosed with autism spectrum disorder (ASD) have increased considerably in the last two decades, with current estimates ranging between 1 and 3% in high income countries (Centers for Disease Control and Prevention 2018; Idring et al. 2015). In addition, while most cases of ASD, particularly milder cases, are diagnosed during later childhood to adolescence (Dal et al. 2013), the mean age at diagnosis has decreased in recent years (Daniels and Mandell 2014). Early diagnosis in ASD is desirable as it makes possible early intervention which can facilitate better outcomes if the interventions are of high quality (Eldevik et al. 2012; Flanagan et al. 2012; Matson and Konst 2014; National Autism Center 2015; Perry et al. 2013; Warren et al. 2012; Wong et al. 2015). Still, earlier diagnoses also challenge educational and clinical settings to provide evidence-based intervention programs to young children on the autism spectrum. For example, in Sweden, 92% of all children between 3 and 4 years attend preschool (National Center for Education Statistics [NCES] 2017) and in Stockholm County, the number of young children receiving treatment for ASD doubled between 2011 and 2016 (Kosidou et al. 2017). To date, there are no tools available for assessing the quality of preschool programs for children with ASD. In the United States, researchers have designed a tool to assess program quality for children with ASD, the Autism Program Environment Rating Scale (APERS, Odom et al. 2018). The purpose of the current study was to appropriately adapt this scale to the prerequisites of the Swedish preschool education system context and to examine its utility and content validity.

In Sweden as in Norway, publicly funded habilitation programs offer intervention for young children with ASD in community-based settings. To conduct the intervention, the municipalities employ preschool staff to provide intervention under the supervision of clinical experts from the health care habilitation centers. Both Norway and Sweden are sparsely populated, with sometimes large geographical distances between habilitation centers and preschools. To assure intervention quality in such a delivery model is challenging. For instance, techniques from the field of applied behavior analysis are often used and are typically unfamiliar to preschool staff in Sweden, who often have negative attitudes towards behavior therapy (Engstrand and Roll-Pettersson 2014; Långh et al. 2017). There are national recommendations (Föreningen Sveriges Habiliteringschefer 2012) concerning early autism intervention in Sweden. However, these do not define the characteristics of a high quality learning environment. Furthermore, there are no national guidelines requiring the use of standardized tools to monitor the quality of the preschool environment in terms of the physical set-up, learning climate, and competence of preschool staff.

Using observational assessments to collect data on classroom and teacher variables that contribute to education quality is well-established in preschool research (Pianta et al. 2005; Reszka et al. 2012; Sam et al. 2015; Westman Andersson et al. 2013). Several assessments of general early childhood education environments, such as the Early Childhood Environment Rating Scale-Revised (Harms et al. 2005; Kärrby 1989; Kärrby and Giota 1994), and the Inclusive Classroom Profile (Lundqvist et al. 2016; Soukakou 2012; Soukakou et al. 2014) are examples of scales that have been developed and translated to Swedish. However, there is to date no instrument available in Swedish designed to assess the quality of the intervention program setting for preschool children with ASD.

The Autism Program Environment Rating Scale-Preschool/Elementary School (APERS) is a rating scale that the U.S. National Professional Development Center on Autism Spectrum Disorder (NPDC) (Odom et al. 2013) designed to assess program quality for children and youth with ASD attending preschool and primary school. NPDC investigators used the information provided by the APERS to give feedback to school program staff about the strengths and weaknesses of their program. School level staff then used the information to develop action plans for program improvement. NPDC staff also used the information to evaluate changes in program quality across time (Odom et al. 2013).

The APERS has two formats, one for preschool and elementary programs (APERS-PE) and one for middle school/high school programs. The APERS-PE, which was utilized in this study, consists of 59 behaviorally anchored items, which are rated on a 5-point Likert-scale. The items are organized into 10 domains, with each having subdomains. In the NPDC professional development work, the APERS was used in 132 school intervention programs in 12 states in the USA (Odom et al. 2013), and it has also been used in Poland, Saudi Arabia and Bangladesh. From their studies in the United States, Odom et al. (2018) reported the instrument to show high internal consistency (Cronbach Alphas > .94) for the total scale and moderate consistency for its subdomains (Cronbach Alphas averaging .70). In addition, an exploratory principal component analysis revealed that APERS items predominantly loaded on a single factor that could be best interpreted in terms of intervention setting quality. Also, the instrument demonstrated sensitivity to improvements in intervention setting quality following professional development provided to teachers in schools.

The APERS was developed in accordance with the U.S. educational context. There are several notable differences between the U.S. system on one hand, and the Swedish and the other Scandinavian countries’ preschool educational systems for children with ASD, on the other, which highlight the need of cultural adaption (Sousa and Rojjanasrirat 2011). For example, Swedish preschools, in comparison with the U.S., places much more emphasis on learning and development through free play rather than structured classroom activities. Moreover, in Swedish preschools individual education plans are not mandatory for children with special needs, as they are in the U.S. Furthermore, the physical preschool environment in Swedish preschool is also arranged differently, with children having free access to several different rooms at most times, and spending substantial time outdoors. Owing to these differences, the APERS may need to be adapted for the Swedish context in order to be utilized as a meaningful assessment of autism learning environment quality in preschools in Sweden. Therefore, the goal of this study was to adapt the APERS-PE for use with preschool programs in Sweden in which children with ASD are enrolled, and to evaluate the content validity of that adaptation.

## Method

### Instrument

The APERS-PE is a rating scale designed to examine the preschool/elementary school teaching environment of children with ASD. The original APERS-PE consists of 59 items, scored on a 5-point Likert-scale from 1 (poor quality) to 5 (high quality), which are grouped into 10 domains and 33 subdomains. The domains are: Learning Environments, Positive Learning Climate, Assessment and Individual Education Plan (IEP) Development, Curriculum and Instructions, Communication, Social Competence, Personal Independence, Functional Behavior, Family Involvement, and Teaming (see Table 1, for all 33 subdomains). It yields a global score for program quality, as well as separate scores for each item, subdomain and domain. APERS-PE is designed to assess both self-contained and inclusive teaching environments. It can be used as a baseline measure of program quality, but also as a follow-up of potential change in program quality. Professionals with expertise on ASD who have undergone training by an experienced APERS rater are authorized to administer the scale.

**Table 1:** Mean CVI (max. 1.00), and mean values (max. 3.00) of experts’ ratings on relevance for subdomains to their domain within parenthesis

Domain	Subdomain	Items	CVI subdomains relevance (and *M*)	CVI item clarity (and *M*)	CVI item comprehensiveness (and *M*)
Total scores		1–56	.99 (2.88)	.98 (2.78)	.97 (2.82)
Learning environments		1–8	.98 (2.84)	.96 (2.57)	.95 (2.66)
	Safety	1–2	.89 (2.67)	.89 (2.44)	1.00 (2.61)
	Organization of Learning Environment	3–4	1.00 (2.56)	.95 (2.44)	.95 (2.56)
	Materials	5–6	1.00 (3.00)	.95 (2.61)	.95 (2.78)
	Visual schedules	7	1.00 (3.00)	1.00 (2.89)	1.00 (3.00)
	Transitions	8	1.00 (3.00)	1.00 (2.67)	.78 (2.33)
Positive learning climate		9–12	1.00 (2.89)	1.00 (2.92)	1.00 (2.89)
	Staff-student interaction	9–10	1.00 (2.89)	1.00 (3.00)	1.00 (2.95)
	Staff behaviors	11	1.00 (3.00)	1.00 (2.78)	1.00 (2.78)
	Promoting diversity	12	1.00 (2.78)	1.00 (2.89)	1.00 (2.89)
Assessment and IEP development		13–18	.95 (2.75)	1.00 (2.74)	.93 (2.81)
	Assessing student progress	13	.89 (2.78)	1.00 (2.33)	.89 (2.56)
	Assessment process	14	.89 (2.67)	1.00 (2.89)	.89 (2.78)
	IEP goals	15–17	1.00 (2.78)	1.00 (2.89)	1.00 (2.93)
	Transition planning	18	1.00 (2.78)	1.00 (2.56)	1.00 (2.78)
Curriculum and instruction		19–30	1.00 (3.00)	.98 (2.80)	.96 (2.81)
	Classroom instruction	19–30	1.00 (3.00)	.98 (2.80)	.96 (2.81)
Communication		31–34	1.00 (3.00)	1.00 (2.89)	.97 (2.94)
	Communication rich environment	31	1.00 (3.00)	1.00 (3.00)	1.00 (3.00)
	Individualized communication instruction	32	1.00 (3.00)	1.00 (2.89)	1.00 (3.00)
	Responsiveness to student communication	33	1.00 (3.00)	1.00 (2.78)	.89 (2.78)
	Communication systems	34	1.00 (3.00)	1.00 (2.89)	1.00 (3.00)
Social competence		35–38	1.00 (2.89)	1.00 (2.83)	1.00 (2.94)
	Arranging opportunities	35–36	1.00 (2.89)	1.00 (2.83)	1.00 (3.00)
	Teaching and modeling	37	1.00 (3.00)	1.00 (2.78)	1.00 (2.78)
	Peer social networks	38	1.00 (2.78)	1.00 (2.89)	1.00 (3.00)
Personal independence and competence		39–41	1.00 (2.78)	1.00 (2.89)	1.00 (2.85)
	Personal independence	39–40	1.00 (2.89)	1.00 (2.94)	1.00 (2.94)
	Self-management	41	1.00 (2.67)	1.00 (2.78)	1.00 (2.67)
Functional behavior (interfering and adaptive)		42–46	1.00 (2.94)	.93 (2.67)	.93 (2.73)
	Proactive strategies	42	1.00 (3.00)	.89 (2.67)	.78 (2.44)
	Behavioral assessment	43–44	1.00 (2.89)	.95 (2.72)	.95 (2.72)
	Behavior management	45	1.00 (3.00)	.89 (2.33)	1.00 (2.89)
	Data collection	46	1.00 (2.89)	1.00 (2.89)	1.00 (2.89)
Family involvement		47–49	1.00 (2.96)	1.00 (2.85)	1.00 (2.96)
	Teaming	47	1.00 (3.00)	1.00 (2.78)	1.00 (2.89)
	Communication	48	1.00 (3.00)	1.00 (2.89)	1.00 (3.00)
	Parent teacher meetings	49	1.00 (2.89)	1.00 (2.89)	1.00 (3.00)
Teaming		50–56	1.00 (2.88)	1.00 (2.83)	1.00 (2.83)
	Team training	50	1.00 (2.67)	1.00 (2.44)	1.00 (3.00)
	Team membership	51–53	1.00 (2.89)	1.00 (2.93)	1.00 (2.89)
	Team meetings	54–55	1.00 (2.89)	1.00 (2.94)	1.00 (3.00)
	Implementation	56	1.00 (2.78)	1.00 (2.67)	1.00 (2.67)

Mean CVI, (max. 1.00) and mean values (max. 3.00) of the clarity and comprehensiveness of items aggregated to subdomain and domain level within parenthesis. *M* = mean

The APERS-PE assessment is comprehensive and usually requires about 6–7 h to administer. The rater gathers available information about the preschool program through observations and collects field notes about program activities in which the children with ASD participate, reviews IEP’s, and interviews parents and teachers/preschool staff. The rater thereafter combines the information and uses it to rate the APERS-PE items.

### Translation and Cultural Adaptation

The process of translating, modifying and assessing the content validity of the Swedish version of APERS-PE was conducted in a multi-step fashion. First, following consent from the original APERS-PE authors (NPDC; Odom et al. 2013) the entire scale was translated from English to Swedish by a Swedish PhD level clinician, fluent in English, experienced in ASD, knowledgeable about the Swedish preschool and early intervention system, and having expertise in scale development, adaptation, and psychometrics. This translator had the target language Swedish as his mother tongue. Second, the authors of the current article, two of which having been trained in the U.S. to administer the APERS, compared the translated scale to the original scale (Sousa and Rojjanasrirat 2011), provided internal feedback on the appropriateness of the translation, as well as the contents of each item. Based on these steps, and using guidelines provided by the original authors on cultural adaption of the instrument (Odom et al. 2013), the authors modified and reduced the Swedish version of APERS to solely focus on preschool settings. This is in contrast to the original version, which was designed for both preschool and elementary school environments. The rationale for this decision was that preschools and elementary schools within the Swedish educational system have substantially different curricula and organizational features. In the adaption process, the official Swedish preschool curriculum guidelines (Skolverket 2016) were used as a reference for adapting contents to the Swedish preschool system. Third, five independent and external ASD preschool and early intervention experts provided feedback on all aspects of the resulting scale. Their feedback was used to further adapt the APERS-PE in accordance with the Swedish preschool contexts. Thereafter, a final Swedish draft, now titled the APERS-P-SE, was generated. In the course of this stepwise procedure, three original items in the domains of Learning Environment (item 2), Personal Independence and Competence (item 40), and Teaming (item 59), respectively, were omitted, as they were consistently deemed to be of non-relevance to the Swedish preschool system. Moreover, modifications in wording and precise contents were made to several items, mainly in the domains of Learning Environment, Family Involvement and Teaming.

### Content Validity

Content validity describes the extent to which the components of an instrument represent and are relevant to the construct for its assessment objective (Haynes et al. 1995). Surprisingly, few diagnostic or intervention instruments in autism have ever been explicitly evaluated for content validity. Mostly, content validity is simply assumed based on face validity or because the scale in question operationalizes established methods (e.g. diagnostic guidelines). Still, ensuring content validity is essential when constructing or adapting a measurement tool, and usually includes evaluations of multiple experts to ensure relevance, representativeness and feasibility of the different elements of the targeted assessment tool (Rubio et al. 2003). Recommendations about the minimum number of content experts needed vary in the literature. Lynn (1986) recommend a range between three and ten experts, while others such as Rubio et al. (2003) recommend a range between 6 and 20 experts. To establish content validity in the current study, nine independent experts assessed the final draft of APERS-P-SE. Inclusion criteria for the experts were: (1) having extensive experience supervising intervention programs for children with ASD and/or other disabilities, (2) being a professional within the Swedish preschool context and potential APERS-P-SE user, and/or (3) being a policy maker within the same area. Experts were drawn from habilitation centers, preschools and other relevant educational and clinical constituents (e.g. The National Agency for Special Needs Education). Four had a PhD degree, and four were Board Certificated Behavior Analysts. Five experts were naïve to the APERS-PE, meaning that they had neither been provided with any information about the cultural adaption process of the instrument nor had previously been in contact with the original English language version. The remaining four were familiar with the English original to varying degrees but were not experienced users. Owing to the comprehensiveness of reviewing the Swedish APERS-P-SE, expert raters were offered a compensation of $150 for their participation in the study.

Prior to evaluating content validity, experts were provided with a written summary of the study’s aims and methods and a description of how to assess content validity (i.e., relevance, clarity, comprehensiveness) of the Swedish APERS-P-SE for the given purpose of evaluating an environment facilitating a positive and effective learning experience for young children with ASD. The experts then rated each item’s level of clarity as well as comprehensiveness on a 4-point Likert-scale ranging from 0 to 3 with “0” indicating that an “item is not clear/item is not comprehensive”, “1” that an “item needs major revision to be clear/item needs major revision to be comprehensive”, “2” that an “item needs minor revisions to be clear/item needs minor revisions to be comprehensive”, and “3” that an “item is clear/item is comprehensive”. Experts also rated each subdomain’s relevance to its superordinate domain (e.g., Safety to Learning Environment), and the relevance of each domain (e.g., Learning Environment) to the whole scale. The rating was on a 4-point Likert scale, with “0” indicating that “subdomain/domain is not relevant”, “1” indicating that “subdomain/domain is not relevant without revision of”, “2” that “subdomain/domain is relevant but needs minor revision” and “3” that “subdomain/domain is very relevant”.

Furthermore, experts rated six statements concerning their beliefs about usefulness, relevance, need, necessity, practical use and personal preference of APERS-P-SE as a whole on a scale from 0 to 3 where “0” was “I do not agree”, “1” was “I agree to some extent”, “2” was “I agree to a large extent” and “3” was “I completely agree”. An example of statement was: “I believe that there is a need for a rating scale such as APERS-P-SE to assess program quality for children with ASD in the Swedish preschool”. Finally, experts were asked to provide written feedback if possible, in order to explain and elaborate the reasons for their ratings.

### Content Validity Index

The Content Validity Index (CVI) is a procedure to quantify content validity. It is commonly computed based on experts’ ratings of an instrument’s relevance or representativeness, and sometimes clarity and/or comprehensiveness, relative to the targeted measurement construct (Davis 1992; Lynn 1986; Rubio et al. 2003; Sousa and Rojjanasrirat 2011). In the current study, CVI was calculated in accordance with established recommendations (Rubio et al. 2003), based on the 4-point Likert scale scores described in the previous section. CVIs were first calculated for APERS-P-SE items, by counting the number of experts who rated items’ level of clarity and comprehensiveness as “2” or “3”, and dividing by the total number of experts, thus providing the proportion of experts evaluating each item as clear and comprehensive. CVI was then calculated for the whole scales’ clarity and comprehensiveness by averaging mean CVI across items. Secondly, CVIs were calculated for subdomains by counting the number of experts who rated subdomain’s relevance for its superordinate domain as “2” or “3”, and dividing by the total number of experts. CVIs were then calculated for all subdomain’s relevance for superordinate domains by averaging mean CVI across all subdomains. Finally, CVIs were calculated for domains by counting the number of experts rating domains relevance for the whole scale as “2” or “3”, divided by the total number of experts. Similarly, CVIs were then calculated for all domain’s relevance for the whole scale by averaging mean CVIs across all domains.

According to the CVI classification outlined by Lynn (1986), which was used to interpret our findings on item, subdomain and domain level, a CVI threshold of ≥ .78 (at least seven out of nine raters scoring item “2” or “3”) is deemed an adequate level of content validity. This threshold is derived from calculating the proportion of experts agreeing on content validity out of the total number of experts, and then determining the standard error of proportion to identify the cut-off level for real versus chance agreement.

In addition to calculating the proportion of experts rating items/subdomains/domains as “2” or “3” (CVIs), mean ratings were calculated for clarity and comprehensiveness on item level, and for relevance on subdomain and domain level. Finally, we also calculated mean values for experts’ ratings for statements about the whole scale (e.g. “Is there a need for a scale such as APERS-P-SE”) by dividing the total score of each of the six statements by the total number of experts.

## Results

### Content Validity

#### Content Validity Index

CVIs for items’ clarity and comprehensiveness, and for subdomains relevance to their superordinate domains are shown in Table 1. CVIs for domain’s relevance to the whole scale are shown in Table 2. Experts’ ratings of items’ clarity and comprehensiveness varied between 1 and 3. All single APERS-P-SE items received a CVI equal to or  above the validity threshold of .78 for clarity and comprehensiveness. Mean CVIs for all items’ level of clarity was .98 while the mean CVI for all items’ level of comprehensiveness was .97.

**Table 2:** CVI values (max. 1) and mean ratings of domains’ relevance (max. 3) for the whole scale

Domain	CVI	Relevance
Learning environments	1.00	2.78
Positive learning climate	1.00	3.00
Assessment and IEP development	1.00	2.89
Curriculum and instruction	1.00	3.00
Communication	1.00	3.00
Social competence	1.00	3.00
Personal independence and competence	1.00	2.89
Functional behavior (interfering and adaptive)	1.00	3.00
Family involvement	1.00	3.00
Teaming	1.00	3.00
Total score	1.00	2.96

Experts’ ratings of subdomain’s relevance for superordinate domain varied between 1 and 3. All subdomains were rated as relevant for their domains and above the threshold for validity with CVIs ranging from .89 (Safety, Assessing Student Progress, Assessment Process) to 1.00 (the other 30 subdomains). Mean CVI over all subdomains was .99 (see Table 1). All APERS-P-SE domains were rated as relevant for operationalizing the evaluation assessment of program environment quality for children with ASD in Swedish preschool (all CVIs = 1.00).

#### Item Scores for Clarity and Comprehensiveness

Mean ratings on items’ clarity (see Table 1) was 2.78 (range 2.22–3.00), and mean ratings on items’ comprehensiveness was 2.82 (range 2.33–3.00). Experts’ ratings on items averaged on domain level showed that the domain Learning Environments displayed the least high rating on both clarity (*M* = 2.57) and comprehensiveness (*M* = 2.66). The highest mean rating of items’ clarity on domain level was for the domain Positive Learning Climate (*M* = 2.92), while the highest mean rating of items’ comprehensiveness on domain level was found for the domain Communication (*M* = 2.94).

#### Subdomain Scores for Relevance

Mean rating of all subdomains relevance to their domain (see Table 1) over all experts was 2.88 (range 2.55–3.00). The subdomain Organization of Learning Environment yielded the least high ratings on relevance to its domain (Learning Environment, *M* = 2.56), while several subdomains such as Transitions were rated as “very relevant” for their superordinate domain by all experts (*M* = 3.00).

#### Domain Scores for Relevance

Mean rating of all domains relevance for operationalizing the overall intervention program quality for children with ASD in Swedish preschool was 2.96 (range 2.78–3.00) (see Table 2). The domain Learning Environments yielded the least high score (2.78), while the domains Assessment and IEP Development, and Personal Independence and Competence each yielded a mean relevance score of 2.89. All other domains were rated with the highest possible level of relevance across all expert raters (3.00).

#### Overall Ratings of APERS-P-SE

The mean of all six ratings of the APERS-P-SE’s overall quality to assess ASD intervention environment was 2.65 (range 2.11–3.00) (see Table 3). The rating concerning the need for a scale such as APERS-P-SE and the previous lack of a scale to assess program quality for children with ASD in the Swedish preschool yielded the highest scores (*M* = 3.00). The rating which yielded the least high score (*M* = 2.11) concerned whether it was realistic to practically use APERS-P-SE in the Swedish preschool to rate program quality.

**Table 3:** Mean values (max. 3) and standard deviations of experts’ ratings on whole scale statements

Statement	Experts’ ratings
1. I believe that APERS-PE-SE is a relevant scale for rating program quality for children with ASD in the Swedish preschool	2.56 (0.53)
2. I believe that APERS-PE-SE is a useful scale to rate program quality for children with ASD in the Swedish preschool	2.56 (0.53)
3. I believe that there is a need for a rating scale such as APERS-PE-SE to rate program quality for children with ASD in the Swedish preschool	3.00 (0.00)
4. I believe that it is realistic to practically use APERS-PE-SE to rate program quality for children with ASD in the Swedish preschool	2.11 (0.60)
5. I regard APERS-PE-SE as a scale that I would like to use to rate program quality for children with ASD in the Swedish preschool	2.67 (0.50)
6. I believe that there has been a lack of a good rating scale to rate program quality for children with ASD in the Swedish preschool	3.00 (0.00)

### Formative Feedback

Experts provided anecdotal formative feedback in their written comments, which was subsequently incorporated into the final version of the APERS-P-SE. One example was about the subdomain “Transitions” (item 8). Several experts questioned the feasibility of teaching preschool age children to always be prepared for unexpected transitions such as fire alarms and power outages: “How do you prepare a child to be prepared for unexpected transitions?” or “Can a child with autism always be prepared for unexpected transitions?”. Suggestions concerning how to improve items’ level of clarity and comprehensiveness included: “Provide examples using ‘free play’ in items addressing transitions as well as unstructured periods of time” and “Please define what is meant by ‘expert’”. Feedback was also provided concerning individual subdomains, domains as well as regarding the six statements on the scale as a whole: “The scale would improve with some minor revisions and adjustments to the Swedish preschool” and “The bar is set high with this scale! Most preschools would receive very low ratings but it might inspire them to improve their work”. In general, lower ratings on item level tended to generate more feedback on improvement. Subsequently, most written comments concerned items in the domains of Learning Environment and Personal Independence where minor revisions were found necessary among some of the experts to increase item clarity, comprehensiveness and general relevance for the Swedish preschool system. Thus, where there was formative consensus among experts about improvements, additional revisions were made to the items, resulting in a final version of the APERS-P-SE.

Experts’ comments about the statements on the entire scale mainly addressed the usefulness of the scale, and the need for a scale such as APERS-P-SE. Several experts pointed out that the scale might be too comprehensive and labor intensive to use as intended, and that the scale places new and high demands on Swedish preschools given their view of the current level of intervention program quality for children with ASD: “The scale is comprehensive and many preschools are light-years from this level. But, it is very important, one has to start somewhere” and “The scale is comprehensive but even more comprehensive is the work that preschools will need to do in order to obtain a high APERS-P-SE rating. Many preschools will not receive high ratings”. Overall, expert raters commented that there is a dire need for a program rating scale such as APERS-P-SE, because Swedish preschools in general do not provide adequate intervention program quality: “There is a need for a scale that can outline the direction for the preschools” and “It would be excellent if this material could be used in the municipalities and in their preschools”.

## Discussion

Valid rating scales of preschool environment quality, in general, and for children with ASD, more specifically, are scarce. Although few countries have a higher percentage of children attending preschool than Sweden (NCES 2017), there is no assessment of the quality of programs delivered in preschools for children with ASD. Preschool intervention programs for children with ASD in Sweden are in need of improved quality control, as previous research on preschool staff has indicated both low levels of knowledge about ASD, and negative attitudes towards specific, individualized interventions (Engstrand and Roll-Pettersson 2014; Långh et al. 2017; Roll-Pettersson et al. 2016). The current study aimed to translate, culturally modify and assess the content validity of the Swedish version of the APERS-PE that has previously demonstrated feasibility and reliability in different international education settings (Odom et al. 2018).

Our results confirm the content validity of the Swedish version of the APERS-PE. Content validity was found at the item, subdomain, domain, and total score levels. The scale was judged to be relevant for the Swedish communities delivering intervention programs for autistic children in community preschools. Also, the results showed the need for quality control in Swedish preschools, and the potential of the APERS-P-SE as a means to ensure adequate quality management. However, the formative feedback provided by the expert panel suggests that the instrument may put high demands on a typical preschool.

During the adaptive process, substantial modifications were needed to fit the original APERS-PE to Swedish conditions. For instance, the APERS-P-SE version only focuses on preschool aged children, as preschool and school teaching conceptually differ in Sweden, not justifying an integrated scale for both settings. The latter highlights conceptual divergence between the educational systems in the USA as compared to Sweden. For example, Swedish preschools emphasize learning through play rather than through structured classroom activities (Skolverket 2016), and individual education plans are not mandatory for children with special needs. Therefore, culturally informed adjustments were deemed necessary in order for APERS-PE to be meaningful in the Swedish context. Adaptations were also made regarding the preschool involvement of experts such as behavior analysts, language-speech therapists, occupational therapists, special educators, and psychologists, as these professions are typically not represented in Swedish preschools, but rather act as external supervisors. Staff at Swedish preschool typically are preschool teachers and child carers, paraprofessionals providing direct instruction to the child with ASD, and, much less frequently, special teachers, music or drama pedagogues. Due to a high fluctuation and lack of sufficiently educated personnel, preschools also employ a significant number of relatively untrained or unexperienced staff (Långh et al. 2017).

There are several limitations to this study. With permission of its original authors, owing to its glossary character and comprehensiveness, the Swedish adaptation of the APERS was not back-translated. The first and second author of this study, both from Sweden, had been trained in the USA to conduct the APERS-PE and achieved consensus reliability. They both reviewed thoroughly the Swedish translation and compared it to the original scale, but it is possible a back translation could have produced a more accurate language translation of the instrument.

With the exception of content validity, other psychometric properties of the Swedish APERS were not examined here. Although we found satisfactory to good consistency between experts rating content validity, additional psychometric studies of the APERS-P-SE [e.g. for reliability (test–retest) as well as validity (construct [convergent, divergent], concurrent, predictive) would be desirable and could be directions for future research. Still, establishing a content-validated cultural adaptation is a necessary prerequisite to meaningfully examine reliability and other forms of validity of the Swedish version of the APERS-PE. In addition, careful cultural adaptation is rarely practiced, and rather translation only is the rule. This study comprehensively examined content validity of the APERS-P-SE using the ratings of experts working in naturalistic settings with broad and in-depth expertise of the area, with various professional backgrounds, and also conducted necessary cultural modifications of the scale resulting in the APERS-P-SE.

Although not necessary a limitation, the APERS-P-SE is labor-intensive, as are the original U.S. American versions. Assessing the quality of a program requires observation in most or all aspects of a program’s scheduled activities, and gathering information from multiple sources. The information is necessary for rating each APERS-P-SE item. This comprehensive level of evaluation is different from coding fidelity of an individual intervention or practices that might occur in specific locations in a class or program (e.g., discrete trial training, dialogic reading). However, such breadth of assessment is necessary in the APERS to generate meaningful information that is likely to have an impact on overall program quality.

We adapted the APERS to the Swedish context, thus a limitation to the significance of this study is that it is uncertain how well it would fit other programs in Scandinavian countries. For example, in Norway there are programs that implement early intensive behavioral intervention in community-based preschool classrooms (Eikeseth et al. 2002; Eldevik et al. 2012), which is similar to the service model conducted in Sweden. While in Denmark and Finland the implementation of behavioral interventions programs for preschool children with ASD in community-based preschools is less frequent, the APERS-P-SE may still be applicable as it assesses the general features of the program that serve as the foundation for individualized intervention practices, which could have different theoretical orientations. Certainly the utility and appropriateness of the Swedish APERS adaptation for other national contexts in Scandinavia remain unanswered and a question of future research.

In conclusion, the APERS-P-SE is a comprehensive rating scale for program environment quality for young children with ASD. It demonstrated high content validity and relevance for improving practice in the Swedish preschool system. Although, the anecdotal feedback from experts suggests that developing a shortened and simplified version of the APERS-P-SE might be indicated, which might be easier to implement, this could compromise the validity of the instrument. Further psychometric studies of the instrument are needed to better understand its strengths, weaknesses and areas of further improvement. Particularly, in order to be able to fully judge the validity of the Swedish APERS-P-SE, research should address its prognostic validity using longitudinal designs, to examine to which extent the quality of treatment environment is associated with the child’s treatment outcome, the ultimate goal of any quality measure.
